# Individual and Binary Mixture Toxicity of Five Nanoparticles in Marine Microalga *Heterosigma akashiwo*

**DOI:** 10.3390/ijms23020990

**Published:** 2022-01-17

**Authors:** Konstantin Pikula, Seyed Ali Johari, Ralph Santos-Oliveira, Kirill Golokhvast

**Affiliations:** 1 Polytechnical Institute, Far Eastern Federal University, 10 Ajax Bay, Russky Island, 690922 Vladivostok, Russia; golokhvast@sfsca.ru; 2 Federal Research Center the Yakut Scientific Center of the Siberian Branch of the Russian Academy of Sciences, 2, Petrovskogo Str., 677000 Yakutsk, Russia; 3 Department of Fisheries, Faculty of Natural Resources, University of Kurdistan, Pasdaran St, Sanandaj 6617715175, Iran; sajohari@gmail.com; 4 Laboratory of Nanoradiopharmaceuticals and Synthesis of Novel Radiopharmaceuticals, Nuclear Engineering Institute, Brazilian Nuclear Energy Commission, R. Helio de Almeida, 75, Rio de Janeiro 21941906, Brazil; presidenciaradiofarmacia@gmail.com; 5 Laboratory of Nanoradiopharmaceuticals and Radiopharmacy, Zona Oeste State University, Av. Manuel Caldeira de Alvarenga, 1203-Campo Grande, Rio de Janeiro 23070200, Brazil; 6 Siberian Federal Scientific Centre of Agrobiotechnology, 633501 Krasnoobsk, Russia; 7 Pacific Geographical Institute, Far Eastern Branch of the Russian Academy of Sciences, Radio 7, 690041 Vladivostok, Russia

**Keywords:** cadmium sulfide, ecotoxicology, flow cytometry, mesoporous silicon dioxide, microalgae bioassay, mixture toxicity, particle agglomeration, titanium dioxide, Trojan horse effect, zinc sulfide

## Abstract

The investigation of the combined toxic action of different types of nanoparticles (NPs) and their interaction between each other and with aquatic organisms is an important problem of modern ecotoxicology. In this study, we assessed the individual and mixture toxicities of cadmium and zinc sulfides (CdS and ZnS), titanium dioxide (TiO_2_), and two types of mesoporous silicon dioxide (with no inclusions (SMB3) and with metal inclusions (SMB24)) by a microalga growth inhibition bioassay. The counting and size measurement of microalga cells and NPs were performed by flow cytometry. The biochemical endpoints were measured by a UV-VIS microplate spectrophotometer. The highest toxicity was observed for SMB24 (EC50, 3.6 mg/L) and CdS (EC50, 21.3 mg/L). A combined toxicity bioassay demonstrated that TiO_2_ and the SMB3 NPs had a synergistic toxic effect in combinations with all the tested samples except SMB24, probably caused by a “Trojan horse effect”. Sample SMB24 had antagonistic toxic action with CdS and ZnS, which was probably caused by metal ion scavenging.

## 1. Introduction

According to existing data [1], the total production of manufactured nanoparticles (NPs) has increased by 5% on average annually since 2000. For example, it has been calculated that the global production of titanium dioxide nanoparticles will reach 2.5 million tons per year by 2025. The Information Handling Services (HIS) Markit Chemical Economics handbook on silicates and silicas in 2017 reported a yearly global consumption of more than 4 million tons of manufactured silica nanoparticles [2].

Manufactured nanomaterials enter the aquatic environment during their production and application as a result of emissions and discharges during environmental cleanup or as waste [3]. In the marine environment, their further transformation (aggregation, deposition, decomposition, transformation, and biotransformation) depends on many environmental factors (e.g., temperature or salinity), as well as the characteristics and properties of the nanoparticles themselves (e.g., type, shape, size, and specific surface area) [4,5]. The processes and mechanisms of influence of carbon, silicon, metal, and metal-containing nanoparticles on marine biota are not quite clear. In particular, the probability and degree of their movement between different trophic levels, the mechanisms of toxic effects on aquatic species, bioaccumulation, and biotransformation are not determined. To maintain the purity of the marine environment, it is important to scientifically determine the risks of new types of technogenic pollutants, such as NPs toward aquatic species. The existing research works devoted to the toxic impact of different types of NPs on microalgae were comprehensively reviewed by Tang et al. (2018) [6]. Our previous work [7], many studies of Gambardella et al. [8,9,10], and the research works of other groups [11,12] were focused on the impact of NPs on sea urchins. The impact of NPs on marine bivalves was studied by the research group of Canesi [13,14,15,16] and the other scientists [17,18,19]. The toxicity of NPs in fish and fish cell lines was reviewed by Cazenave et al. (2019) [20]. It should be noted that one of the hottest issues in nanotoxicology is the development of predictive models for the risk assessment of NPs [21,22,23,24]. The toxicity of NPs in combinations with chemicals [25], metallic pollutants [26], natural organic matter [27], other pollutants, and environmental substances [28] are some of the major concerns of environmental nanotoxicology. Another important problem is the lack of understanding of the mechanisms of interaction between different NPs. The existing models of chemical mixture effects include non-interactive action (concentration addition or independent action) and interactive action (synergism or antagonism) [29]. However, the existing predictable models cannot be applied to the variety of NPs with different properties and volatile behavior in the environment.

The knowledge about the environmental behavior of NPs and the interaction of metal-based NPs with aquatic organisms is summarized in a review by Peng et al. (2017) [30]. It should be highlighted that the aggregation or dissolution of NPs and reactive oxygen species (ROS) generation strongly depend on the size, shape, hydrophobicity, temperature, pH, salinity, concentration, and other parameters [4,31,32]. These processes can dramatically change the physiochemical properties, bioavailability, and toxicity of NPs [33,34]. Considering this, the understanding of the environmental behavior and toxicity of NPs requires experiments in real natural environments, which reflects the diversity of environmental parameters and has no simplifications associated with laboratory conditions. The area of mesocosm experiments in the ecotoxicology of NPs is under development, and there are several interesting research works [35,36]. However, this is a controversial approach. The results of a real nature experiment are difficult to interpret to determine the effect of a specific component or parameter on an obtained result. Therefore, before the realistic environmental study, it is important to determine the effects of isolated parameters and their combinations.

It was reported that the toxic effects of metal-oxide nanoparticles are a result of oxidative stress and membrane damage, provoked by releasing metal ions into the media [37]. However, it was accurately noticed by Deng et al. (2018) that it is probably impossible to fully understand the toxicity mechanism of metal oxide NPs due to the complicated interactions with cellular or media components and uninterpretable outcomes [38]. Silicon dioxide (SiO_2_) is considered a safe NP, having a variety of applications including food additives and drug delivery [2,39]. However, it was reported that a high level of SiO_2_ NPs can cause cytotoxicity, ROS generation, and other health risks in human cells and rats [40,41].

Nowadays, few studies have investigated the combined effects of binary and complex mixtures of NPs on organisms. There are previous studies that used bacteria [42], microalgae [43,44], crustaceans, and fish [45] in the risk assessment of binary mixtures of NPs. In general, the impact of suspended particles or dissolved metal ions and the interaction between mixtures of NPs and aquatic organisms of different trophic levels remains uncertain, and this area requires further study.

This work set out with the aim of assessing the interaction between different types of NPs in a microalga bioassay. Microalga was selected as a reliable, sensitive, and wildly used test model [46,47] which represented the initial level of aquatic food chains [48]. In this work, we used the raphidophyte microalga *Heterosigma akashiwo* and five types of NPs. *H. akashiwo* was chosen as a common test species used in toxicity bioassays with different chemicals and substances, including NPs [49,50]. The used NPs were chosen based on their representativeness in the industry of nanotechnology and different expected levels and mechanisms of aquatic toxicity, which represented an interest for a combined toxicity bioassay.

## 2. Results

### 2.1. First Stage of the Bioassay: Single Nanoparticle Assessment

The calculated effective concentrations of the five used NPs, namely cadmium and zinc sulfides (CdS and ZnS), titanium dioxide (TiO_2_), and two types of mesoporous silicon dioxide (SMB3 and SMB24), which caused 10% (EC10) and 50% (EC50) inhibition of microalgal growth, are given in Table 1.

The highest toxicity was observed for SMB24, followed by CdS, ZnS, and TiO_2_. The lowest toxic influence on *H. akashiwo* had the sample of the mesoporous silica NPs SMB3.

### 2.2. Second Stage of the Bioassay: Binary Mixture Assessment

The concentrations of the NPs used in the second stage of the bioassay were chosen based on the calculated EC10 values, as given in Table 2.

**Figure 1:**
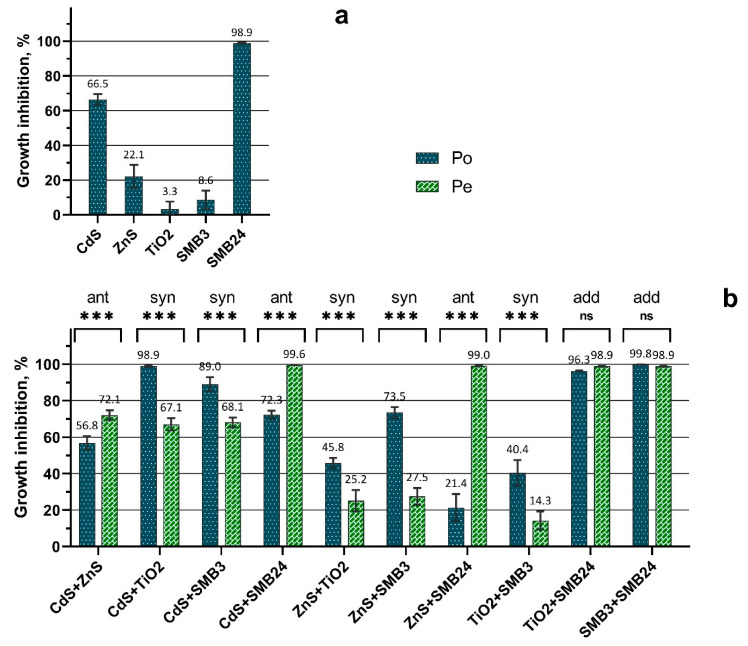
Observed and expected growth inhibition of *H. akashiwo* exposed to single and binary mixtures of NPs. (**a**) Observed toxicity of single NPs. (**b**) Comparison between the observed and expected toxicity of binary mixtures of NPs. P_o_ = observed toxicity (growth inhibition); P_e_ = expected toxicity (growth inhibition); ant = antagonistic; syn = synergistic; add = additive; *** = *p* < 0.0001; ns = nonsignificant (*p* > 0.05). Growth inhibition was calculated compared with the control, where 0% is no observed effect and 100% is the death of all the cells.

The comparison between the observed growth inhibition (P_o_) calculated by Equation (1) and expected growth inhibition (P_e_) calculated by Equation (2) is demonstrated in Figure 1.

The obtained results (Figure 1) demonstrated that the samples of SMB24 and CdS had a significantly higher toxic effect on the growth of *H. akashiwo* than was calculated in the first stage of the bioassay (Table 1). The results of the second stage of the bioassay (Figure 1) demonstrated that the toxic action of binary mixtures of NPs differed between the combinations according to the comparison between the observed (P_o_) and expected toxicity (P_e_). The cases with no significant difference between P_o_ and P_e_, the cases with P_o_ levels significantly higher than the P_e_ levels, and the cases with P_o_ levels significantly lower than the P_e_ levels could be assumed to be additive, synergistic, and antagonistic toxic actions, respectively.

The highest antagonistic action was observed for the combination of ZnS and SMB24 (Figure 1b). Subsequently, the presence of ZnS reduced the high toxicity of SMB24, compared with the effects of single NPs (Figure 1a). The similar but not so intensive antagonistic effect was caused by the presence of CdS NPs in combination with SMB24. The lowest significant antagonistic effect among the tested combinations of NPs was demonstrated by the combination of CdS and ZnS.

The synergistic action was observed for the combinations of CdS and ZnS with TiO_2_ and SMB3, including the combination of TiO_2_ with SMB3. At the same time, the combinations of SMB24 with TiO_2_ and with SMB3 had an additive effect (i.e., both toxicants had independent action).

In general, the mixture toxicity bioassay demonstrated that all the combinations of the used NPs with SMB24 had either antagonistic or additive effects. On the contrary, all the combinations of the used NPs with TiO_2_ and SMB3 had either synergistic or additive effects.

**Figure 2:**
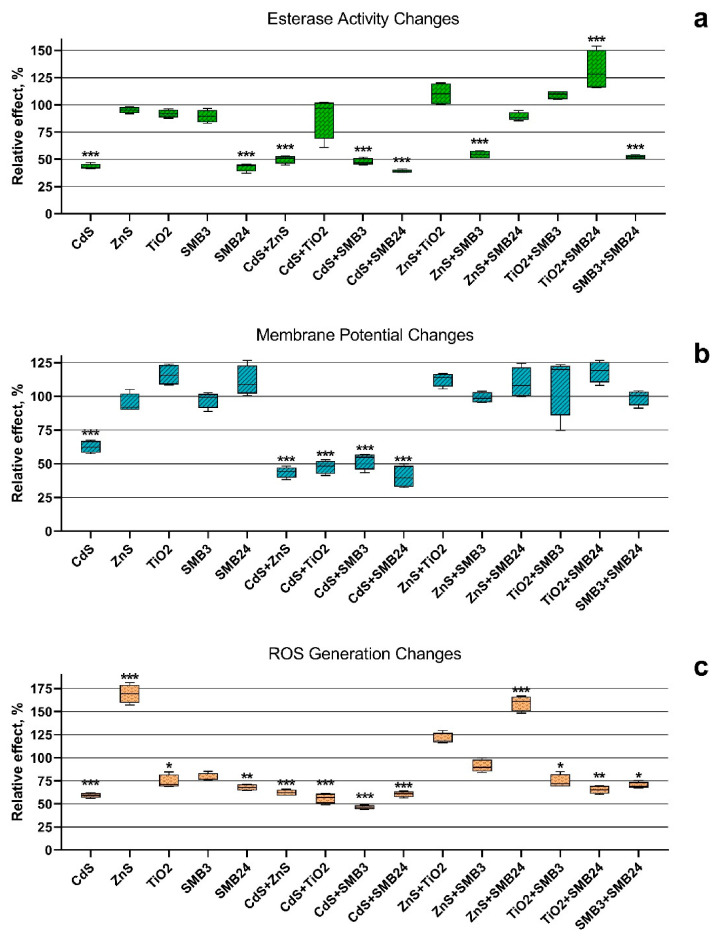
The biochemical changes in the cells of *H. akashiwo* exposed to the single and binary mixtures of the NPs. (**a**) Esterase activity changes. (**b**) Membrane potential changes. (**c**) ROS generation changes. *** *p* < 0.0001. ** *p* < 0.001. * *p* < 0.05.

The changes in esterase activity, membrane potential, and ROS generation in the cells of *H. akashiwo* exposed to the single and binary mixtures of the NPs were calculated by Equation (3) and are represented in Figure 2.

It should be noted that CdS and SMB24, as well as the combinations of all the used NPs with these two samples, significantly reduced the esterase activity of microalga cells (Figure 2a). The exception was observed only for the combination of CdS and SMB24 with TiO_2_. The presence of TiO_2_ almost normalized the esterase activity of *H. akashiwo* exposed to CdS, and the combination of TiO_2_ with SMB24 significantly increased the esterase activity of microalga cells.

The highest esterase activity inhibition caused by a single exposure of CdS and SMB24 (Figure 2a) correlated with the highest observed toxicity of these two samples (Figure 1a). It might be expected that the normalization of esterase activity in the binary exposure of CdS and SMB24 with TiO_2_ should reduce the toxicity by antagonistic action. However, this assumption did not correlate with the binary toxicity data (Figure 1a). Nevertheless, the demonstrated prevention of esterase activity inhibition might facilitate faster adaptation of the cells to the toxic influence. This hypothesis required examination in further chronic toxicity studies.

Significant membrane depolarization (Figure 2b) was observed only for CdS and for all the combinations of the used NPs with CdS. All the other samples did not cause a significant change in microalga membrane polarization. It should be noted that despite similar membrane depolarization (Figure 2b), the binary combinations of the used NPs with CdS revealed both antagonistic and synergistic interaction (Figure 1b). In this case, membrane polarization by itself did not directly influence growth inhibition or the death of microalga cells.

A significant increase in reactive oxygen species (ROS) generation was observed for the microalga cells exposed to ZnS and the mixture of ZnS with SMB24 (Figure 2c). Most of the other samples decreased ROS generation compared with the control. The highest significant decrease in microalgal ROS generation (*p* < 0.0001) was caused by the influence of CdS and the combinations of all the other NPs with CdS. The insignificant influence on ROS generation was observed only for SMB3 and the combinations of ZnS with TiO_2_ and SMB3. It should be highlighted that all the combinations of NPs with ZnS, except for the combination with CdS, had normalized or increased ROS generation compared with the single exposure of microalgae to these NPs. Interestingly, the combination of ZnS with SMB24, which caused the highest increase in ROS generation, demonstrated the highest antagonistic interaction in the growth rate inhibition bioassay (Figure 1b). These data correlated with the previous observation regarding the ability of ZnS to reduce the toxicity of SMB24.

**Figure 3:**
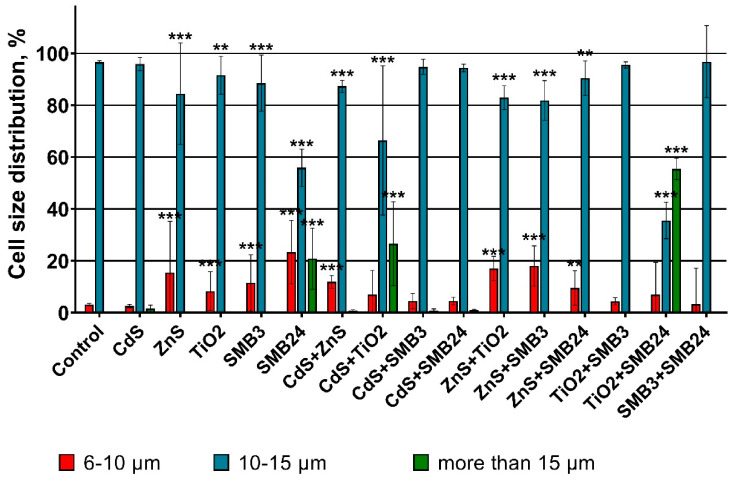
The changes in the size of *H. akashiwo* cells exposed to the single and binary mixtures of the NPs. *** *p* < 0.0001. ** *p* < 0.001.

The influence of single and binary mixtures of the NPs on the size of the microalga cells is represented in Figure 3.

The obtained data demonstrated that the control group had more than 90% *H. akashiwo* cells in the size range between 10 and 15 µm. According to the results, the highest increase in the microalga cell size was caused by the influence of single SMB24 NPs and the binary mixtures of SMB24 with TiO_2_ and CdS with TiO_2_. In these cases, there was a significant (*p* < 0.0001) increase in the number of cells in the size range of more than 15 µm. It should be noticed that single exposure of microalgae to CdS did not show any significant cell size change, and TiO_2_ even caused a decrease in cell size, although the mixture influence of TiO_2_ with CdS increased the size of *H. akashiwo*. In general, all the cases with an increase in the cell size of the microalga correlated with high mortality and growth inhibition (Figure 1). Hence, the enlargement of microalga cells was a consequence of a high toxic influence. However, at the same time, the similar high toxic influence of the combinations of CdS with TiO_2_ and SMB3 with SMB24 did not cause the cell enlargement.

A significant decrease in cell size (an increase of the share of the smaller cells (6–10 µm)) was observed for the samples ZnS, TiO_2_, SMB3, and for the combinations of ZnS with all the other tested samples. These cases correlated with the lower observed toxicity, compared with the cases caused by cell enlargement.

### 2.3. Particle Transformation Assessment

**Figure 4:**
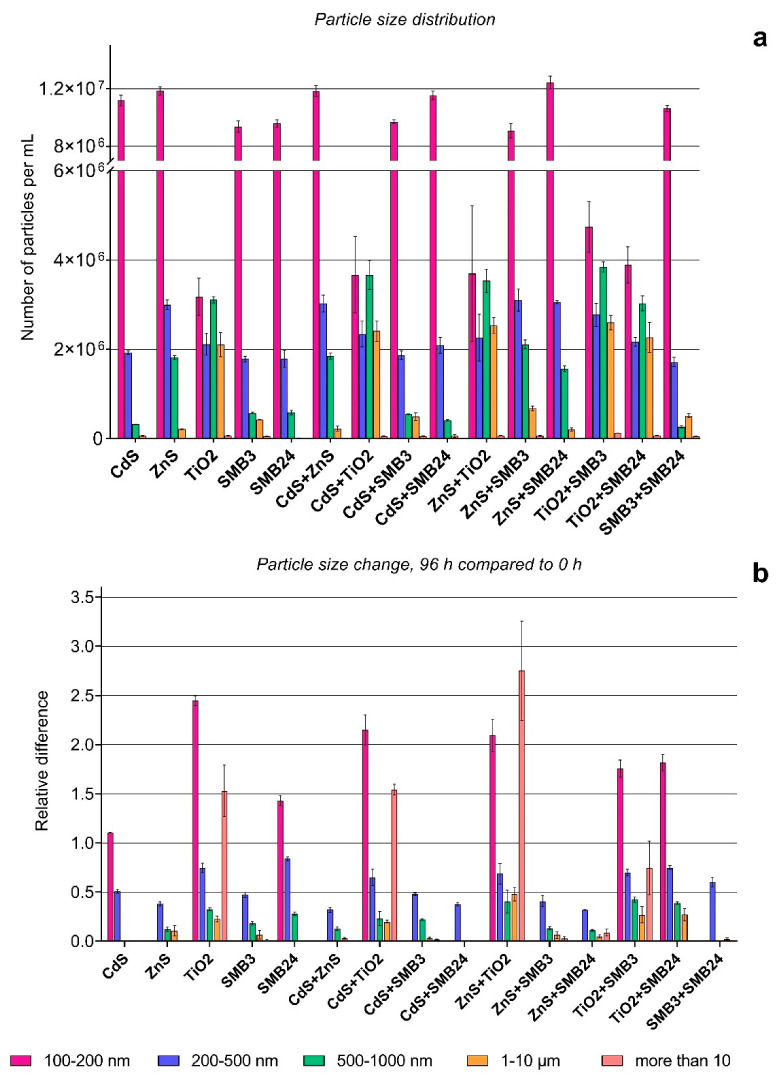
Particle size distribution and size change with time. (**a**) Size distribution of NPs after dispersion. (**b**) Relative particle size change after 96 h compared with 0 h. The values higher than 1.0 mean surpassing the experimental group compared with the reference group, and the values below 1.0 mean a corresponding reduction in the experimental group compared with the reference group (the lower value, the higher the reduction). Statistically insignificant results (*p* > 0.05) are not represented on the graph.

**Figure 5:**
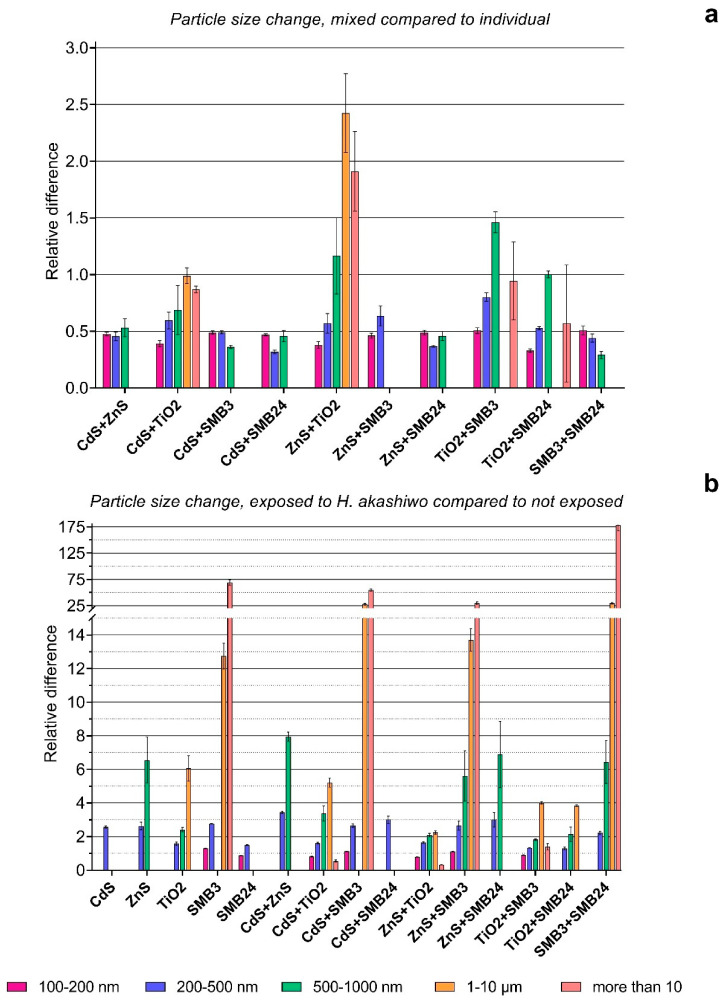
Effects of binary mixtures and presence of *H. akashiwo* on the size of the NPs. (**a**) Relative particle size change in binary mixtures compared with the sum of individual NPs after 96 h. (**b**) Relative particle size change after 96 h of exposure with *H. akashiwo* compared with 96 h of exposure without *H. akashiwo*. The values higher than 1.0 mean surpassing the experimental group compared with the reference group, and the values below 1.0 mean the corresponding reduction of the experimental group compared with the reference group (the lower value, the higher the reduction). Statistically insignificant results (*p* > 0.05) are not represented on the graphs.

The initial particle size distribution and particle size changes after 96 h of exposure are represented in Figure 4. The particle size changes were affected by combinations of NPs in binary mixtures and by the presence of microalga cells after 96 h of exposure as represented in Figure 5. The relative differences in Figure 4b and Figure 5b were calculated by Equation (4). The relative differences in Figure 5a were calculated by Equation (5). The particles smaller than 100 nm are not represented on the graph because of the resolution capacity of the CytoFLEX flow cytometer and the high noise ratio in this size range.

The initial particle size distribution (Figure 4a) demonstrated the highest agglomeration of the used TiO_2_ NPs. The single TiO_2_ sample and all the combinations with the other NPs demonstrated the highest number of particles in the size ranges of 500–1000 nm and 1–10 µm, compared with the other samples and their combinations. At the same time, a single TiO_2_ sample and all the binary combinations with TiO_2_ had the lowest number of particles in a size range of 100–200 nm, which also demonstrates that the TiO_2_ sample facilitated the aggregation of the other NPs. The samples of CdS, ZnS, SMB3, SMB24, and their combinations in the used concentrations had a similarly high number of particles in the size range of 100–200 nm.

Figure 4b shows that the highest relative agglomeration after 96 h compared with the initial size distribution had the combination of TiO_2_ with ZnS, followed by the combination of TiO_2_ with CdS and the single TiO_2_ sample. All these cases demonstrated the same pattern, with an increase in the particles in size ranges of more than 10 µm and 100–200 nm and a simultaneous decrease in the number of particles in the size ranges between 200 nm and 10 µm.

CdS demonstrated a slight increase in the number of particles in the size range of 100–200 nm and a 50% decrease in the particles in the 200–500 nm size range, which might be explained by the dissolution of the aggregated NPs. The samples of ZnS, SMB3, the combinations of CdS and ZnS with the other particles except for TiO_2_, and the combination of SMB3 with SMB24 had no significant difference in the number of 100–200 nm particles, but all these samples demonstrated a decrease in 200–500 nm particles. The samples of ZnS and SMB3 and their combinations with each other and with CdS and SMB24 demonstrated a high decrease in the number of 500–1000 nm particles.

Figure 5a also demonstrated the highest particle agglomeration caused by the combination of TiO_2_ with ZnS, compared with the summation of the measurements of these NPs individually. The combination of TiO_2_ with SMB3 increased the number of 500–1000 nm particles. The other samples in combinations had a lower number of particles compared with the sum of their single measurements, which can mean that interaction between the used NPs supported their faster dissociation compared with single NPs.

Figure 5b demonstrated that the presence of *H. akashiwo* had an extremely high influence on the aggregation of sample SMB3 and all the combinations of the other used samples with SMB3. The increase in the particles in the size range of 1–10 µm of 13–25 times and particles more than 10 µm of 25–175 times for these samples should be assessed, considering the very low number of such particles in the initial suspensions (Figure 4a). It means that the number of particles in the 1–10 µm and more than 10 µm size ranges did not become substantially high, but the influence of *H. akashiwo* cells on the agglomeration of SMB3 was obvious. The presence of the microalga highly increased the number of particles in the size range of 500–1000 nm for ZnS and all the combinations of the other NPs with ZnS. The lowest effect was observed for the samples of CdS, SMB24, and their combination. *H. akashiwo* had either no significant effect or slightly changed the number of 100–200 nm particles compared with the control (relative difference of 1.0) for all the used samples and their combinations.

### 2.4. Summary

The obtained results were summarized in Table 3 to facilitate further the discussion process.

## 3. Discussion

This study focused on the single and combined toxic effects of metal-containing, metal oxide, and silicon oxide NPs on microalgae. Moreover, this research work evaluated the influence of the interaction between different types of NPs and between NPs and microalga cells on the agglomeration of the NPs.

The recent concept suggested considering even pure NPs as a mixture due to their transformation, dissociation, agglomeration with environmental components, and impurities in their composition [38]. In this study, the used mesoporous SiO_2_ NPs SMB24 had inclusions of ZnO and Ag represent strong antimicrobial and cytotoxic properties, according to the manufacturer information (CENNANO Co., Ltd., Ulsan, Korea). This sample of NPs can be considered a mixture by itself because of the impurities. The obtained results of the bioassay correlate with the manufacturer information, as this sample demonstrated the highest toxicity among the tested NPs (EC50, 3.6 mg/L).

We can conclude that the toxic properties of SMB24 were caused directly by the metal impurities of ZnO and Ag, because sample SMB3 with similar structural parameters but without any impurities had the lowest toxicity toward *H. akashiwo* cells among the tested NPs (EC50, 252.8 mg/L). It was reported that ZnO is predominantly present in biological media in ionic form and binds with proteins by electrostatic attraction [51]. Ye et al. (2018) demonstrated in their work that ZnO NPs and graphene oxide NPs had an additive toxic effect in the microalga *Scenedesmus obliquus* and crustacean *Daphnia magna* and had an antagonistic effect in the fish *Danio rerio* [45]. In our work, only the addition of ZnS and CdS decreased the toxicity of SMB24 (Figure 1b) among the other tested NPs. The observed effect might be caused by scavenging of additional Zn^2+^ ions and ZnO inclusions from SMB24, which made the properties of SMB24 closer to the relatively safe SMB3. The ability of ZnO and ZnS to form nanocomposites in water under normal conditions was reported previously [52]. Our results correlate with this conclusion, because the combination of SMB24 with ZnS demonstrated one of the highest dissociations of the aggregated particles in all the size ranges above 200 nm (Figure 4b) and, at the same time, demonstrated antagonistic toxic action. Other important findings of Ye et al. (2018) demonstrated that the main contribution to toxicity was from the particles rather than from ions of Zn^2+^, which were absorbed by graphene oxide NPs [45]. Therefore, the decrease in the number of particle agglomerates could reduce the direct cytotoxicity and mechanical damage of microalga cells produced by agglomerated NPs, and it might be another reason for the antagonistic toxic action.

On the contrary to the case discussed above, TiO_2_ NPs had synergistic toxic action in combinations with all the used samples except SMB24 (Figure 1b). This observation also correlates with the concept of higher cytotoxicity of agglomerated NPs to microalga cells, because the single TiO_2_ sample and the combinations with TiO_2_ demonstrated the highest agglomeration rate of the particles in the size ranges of 1–10 µm and 100–200 nm (Figure 4b). These results can be difficult to discuss because the used concentrations were different between the NPs (Table 2), but despite higher concentrations of ZnS, TiO_2_, and SMB3, the combination of ZnS with TiO_2_ significantly increased the particle size compared with the combination of ZnS with SMB3 (Figure 4b and Figure 5a). This observation correlates with the previous study [53], where the authors described the mechanisms of Zn^2+^ absorption on TiO_2_ NPs in a natural aqueous medium and hypothesized that the toxicity of soluble NPs significantly depends on the presence of more stable NPs, such as TiO_2_ NPs. In our case, we can hypothesize that the synergistic toxic effect might be supported by a “Trojan horse effect” which allows the metal-carrying NPs to cross biological membranes and to be internalized into living organisms [53]. Therefore, TiO_2_ NPs can bind Cd^2+^ and Zn^2+^ ions and increase their toxicity to aquatic species, as was previously reported for the freshwater microalga *Chlamydomonas reinhardtii* [54] and crustacean *D. magna* [55].

Interesting findings were observed for the SMB3 NPs. All the combinations with SMB3 (except the combination with SMB24) also demonstrated synergistic toxic action. In this case, the particles by themselves were dissociated with time in all the combinations (Figure 4b), but the interaction with microalga cells facilitated significant agglomeration of the SMB3 NPs (Figure 5b). The hydrophilic surface of silicon NPs provides their affinity to microalga cells, allows them to easily move in the water body, and increases the possibility of NPs to have a contact with microalga cells [56,57]. Moreover, it was previously demonstrated that the phytotoxicity of silicon NPs increases with an increase in particle size [58]. However, the results of our study do not correlate with the previous work, where the authors demonstrated that the presence of TiO_2_ and SiO_2_ NPs reduced the toxicity of cadmium to *C. reinhardtii* [59]. The authors hypothesized that the effects of NPs on the toxicity of Cd toward microalgae were dependent on the intracellular oxidative stress and the antagonistic toxic effect associated with reduced ROS generation in microalgae. The authors demonstrated that *C. reinhardtii* significantly increased ROS generation after cadmium exposure and normalized it after the SiO_2_ addition [59]. Our study demonstrated that the antioxidative system of *H. akashiwo* responded with a decrease in ROS generation after both Cd exposure and the combined exposure of Cd and SMB3 (Figure 2b). In this case, the antioxidative system of *H. akashiwo* could be already exhausted by Cd exposure, and the high affinity of SMB3 with microalgae cells increased the toxicity. All these observations confirm that the combined toxicity of the NPs was strongly dependent on the used concentrations and species and required further study.

Our study showed that SMB3 had no significant effect on the measured biochemical parameters of the microalga cells, but in all the combinations with the other NPs, the changes in esterase activity, membrane potential, and ROS generation in the microalga cells almost mimicked the corresponding changes caused by the second sample of NPs used in this combination (Table 3). A similar toxic action of silicon nanotubes in four microalgae species, including *H. akashiwo*, was previously reported in our work [49]. However, if the used NPs had different and independent toxic actions with SMB3, we would observe the toxicity addition effect instead of a synergistic one [29]. Considering the synergistic toxic action and absence of biochemical changes, here we can highlight the predominant action of particulate matter toward the toxicity of SMB3. The combinations of SMB3 with the other samples of NPs might also cause a “Trojan horse effect” and provide synergistic toxic action. Consequently, even the relatively safe NPs, such as silicon dioxide SMB3, can be a serious threat to aquatic organisms in combination with other chemicals. The other chemicals do not have to demonstrate high toxic action, as we showed for the combination of SMB3 with TiO_2_ (Figure 1b), but synergistic toxic action might be very dangerous in aquatic species.

In general, the obtained results (Table 3) were not able to provide a clear understanding of the interaction between different NPs and the microalga cells, but the stated findings can highlight the lack of knowledge and drawbacks in the experimental design, which should be considered in further research. In further research, we plan to add the direct measurement of metal ions released in media by ICP-MS. The other directions for further study include the chronic toxicity assessment with lower concentrations of NPs, development of a standard protocol for the assessment of NP mixtures, application of a broader set of aquatic species from different trophic levels, the inclusion of omics technologies to determine the direct toxic pathways of NPs and their combinations, and the assessment of the combined toxic action of the NPs with other chemicals and substances.

## 4. Materials and Methods

### 4.1. Nanoparticles

For the bioassays, we used five samples of NPs (Table 4), namely cadmium and zinc sulfides (CdS and ZnS), titanium dioxide (TiO_2_), and two types of mesoporous silicon dioxide (SMB3 and SMB24).

Particle acquisitions were supported by the team staff of Far Eastern Federal University, working under the state task of the Russian Ministry of Science and Higher Education (project 0657-2020-0013).

### 4.2. Microalga Culture

The culture of a raphidophyte microalga *H. akashiwo* (Raphidophyceae) [61] was provided by The Resource Collection “Marine Biobank” of the National Scientific Center of Marine Biology in the Far Eastern Branch of the Russian Academy of Sciences (NSCMB FEB RAS).

The morphology of *H. akashiwo* was described in detail [61]. However, it should be highlighted that *H. akashiwo* does not have a shell like most of the algae species, but it has amorphous vesicles under the cell wall. *H. akashiwo* has a mixed form of feeding, which includes photosynthesis, direct nutrition uptake, and the eating of bacterium [62].

Culturing of the microalgae and toxicity test conditions were maintained following the guidance of OECD No. 201 [63] with minor modifications (Table 5).

### 4.3. Exposure Protocol

The tested NPs were added to 0.22 µm of filtered seawater to obtain the working suspensions with a concentration of 1000 mg/L. Before each series of bioassays, the working suspensions of the NPs were sonicated with a Bandelin Sonopuls GM 3100 ultrasound homogenizer (Bandelin Electronic GmbH & Co. KG, Berlin, Germany) with a high-frequency power of 100 W for 30 min. The sonication was performed to prevent the initial particle agglomeration according to the protocols of particle suspension testing [64].

All the bioassays were performed in 24-well plates in quadruplicate. The volume of the microalga aliquots in each well was 2 mL. The wells with only f/2 medium [65] were taken as a control group. The duration of microalga exposure to the NPs was 96 h. The salinity and pH were checked before and after exposure by a SevenGo Duo pH/Cond meter SG23-EL-Kit (Mettler Toledo, Switzerland).

The first stage of the bioassay involved single NP testing. Here, we used the concentrations of 10, 25, 50, 100, 125, and 150 mg/L for all the tested NPs. The preliminary results demonstrated that the highly cytotoxic sample SMB24 required reduction of the used concentrations and, therefore, the concentrations of 1 and 5 mg/L were additionally applied for this sample. Based on the results of the first stage of the bioassay, we calculated the effective concentrations of the NPs, which caused 10% (EC10) and 50% (EC50) inhibition of microalgal growth. The calculation of the EC10 and EC50 values was performed by nonlinear regression fitting in GraphPad Prism 8.0.2 (GraphPad Software, San Diego, CA, USA). The control group was taken as 100%.

The second stage of the bioassay was the testing of the binary mixtures of the NPs. Here, we used the EC10 concentrations calculated for each NP sample in the first stage. Hence, the microalga cells were exposed to the calculated EC10 concentrations of each NP sample individually (comparative control) and in binary combinations with the other samples.

To assess the toxic effects of single and binary mixtures on the second stage of the bioassay, the observed toxicity (P_o_) was calculated as a percent of growth inhibition compared with the control of Equation (1):

(1) $$P_{o}=100-\frac{y}{\bar{x}}\times 100$$

where y is the response of the exposure and $\bar{x}$ is the mean of the control response.

Then, the observed toxicity (P_o_) of the binary mixtures was compared with the theoretically expected toxicity (P_e_) calculated by Equation (2) based on probability theory [66]:

(2) $$P_{e\left(1+2\right)}=P_{1}+P_{2}-P_{1}\times P_{2}/100$$

where P_1_ is the mean observed toxicity caused by NP sample N_1_ and P_2_ is the mean observed toxicity caused by NP sample N_2_. The observed toxic effect was considered synergistic or antagonistic if P_o_ was significantly (*p* < 0.05) higher or lower than P_e_, respectively. If there was no significant difference (*p* > 0.05) between P_o_ and P_e_, then the interaction was determined to be additive.

### 4.4. Cell Count, Staining, and Measurement Protocols

Before all the measurements, 100 µL of exposed microalga aliquot from each well of the 24-well plate was transferred into a 96-well plate and stained with specific fluorescent dyes as described below. The used endpoints, biomarkers, and parameters of their registration are represented in Table 6. The excitation source and emission channels were selected according to the maximum emission of the used fluorescent dyes, which were provided by the manufacturer (Molecular Probes, Eugene, OR, USA).

The counting of the microalga cells and the evaluation of their morphological changes were performed with a CytoFLEX flow cytometer (Beckman Coulter, Indianapolis, IN, USA) with the software package CytExpert v.2.0. The determination of the microalga cells in the analyzed samples was carried out using the parameters of the microalga cell size, granularity, and fluorescence of chlorophyll *a* (emission channel: 690 nm) as described previously [67]. Each sample was measured at a flow rate of 50 μL/min for 30 s.

The registration of the biochemical endpoints was performed with a GloMax Multi+ UV-VIS microplate reader E9032 (Promega Corporation, Madison, WI, USA). According to the similar emission maximum of the used dyes, the measurements of all endpoints by the GloMax microplate reader were made as separate independent series. The results were obtained as the mean fluorescent intensity represented in arbitrary units.

The growth inhibition changes of the microalga cells were measured both in the first and second stages of the bioassay. The size, esterase activity, membrane potential, and ROS generation in the exposed microalga cells were registered only on the second stage of the bioassay.

The changes in microalgal esterase activity, membrane potential, and ROS generation were estimated as an observed relative effect (RE, % of control) by Equation (3) [68]:

(3) $$\mathrm{RE}=\frac{y}{\bar{x}}\times 100$$

where y is the response of the exposure and $\bar{x}$ is the mean of the control response.

### 4.5. The Estimation of Particle Size Distribution and Particle Size Changes after Exposure

To assess the size distribution of the used NPs and their size changes after exposure, we used a CytoFLEX flow cytometer in two different working configurations. The particles in the size range of 100–1000 nm were registered by the forward scatter intensity of the violet laser (405 nm) and used a mixture of fluorescent particles Megamix-Plus SSC and Megamix-Plus FSC (BioCytex, Marseille, France). The particles in the size range of 1–10 µm were registered by the forward scatter intensity of the blue laser (485 nm) and size calibration kit (batch F13838, Molecular Probes, USA). All the measurements were performed in these two configurations independently in triplicate. Each measurement was performed at a flow rate of 50 μL/min for 30 s.

The particle size distribution was assessed for the EC10 concentrations of the NPs calculated in the first stage of the bioassay and for all their binary combinations. To assess the changes in the particle sizes with time and to assess the effect of binary combinations on the particle size, the measurements were carried out immediately after the dispersion of single and binary mixtures of NPs and after 96 h of exposure in the same condition as the microalgae bioassay but without adding the aliquot of microalga cells. To assess the impact of the microalga cells on the particle size, the measurements of the particle sizes were performed after 96 h of exposure of the microalga cells with single and binary mixtures of NPs. In this case, the microalga cells were excluded from counting in the CytExpert software by chlorophyll *a* fluorescence in the emission channel of 690 nm.

The changes in the size distribution of the NPs after 96 h compared with 0 h and after 96 h of exposure with *H. akashiwo* compared with 96 h of exposure without the alga were estimated as a relative difference (RD, times) by Equation (4):

(4) $$\mathrm{RD}=\frac{y_{i}}{\bar{x_{i}}}$$

where y*_i_* is the number of particles in a size range i registered in the compared group and $\bar{x_{i}}$ is the mean number of particles in a size range i registered in the reference group.

The relative particle size change in the binary mixtures compared with the sum of the individual NPs after 96 h of exposure was calculated as a relative difference of the mixtures (RD_(1+2)_, times) by Equation (5):

(5) $$\mathrm{RD}=\frac{y_{\left(1+2\right)i}}{\bar{x_{1i}}+\bar{x_{2i}}}$$

where y_(1+2)_ is the number of particles in a size range *i* registered in the binary mixture of NPs after 96 h of exposure and $\bar{x_{1i}}$ and $\bar{x_{2i}}$ are the mean number of particles registered after 96 h of exposure in a size range *i* in single-NP samples N_1_ and N_2_, respectively.

### 4.6. Statistical Analysis

Statistical analyses were performed by GraphPad Prism 8.0.2 (GraphPad Software, San Diego, CA, USA).

In the first stage of the bioassay, the statistical significance of the microalga growth rate’s dependence on the concentrations of the NPs was tested by one-way ANOVA.

In the second stage of the bioassay, multiple Student’s *t*-tests were used to assess the significance of the difference between P_o_ and P_e_, the control and exposed groups in terms of esterase activity, the membrane potential, ROS generation, and algal cell size assessment, the particle numbers in the corresponding size ranges before and after 96 h of exposure, the particle numbers in corresponding size ranges with and without microalga cells, and between the calculated sum of the numbers of particles in the corresponding size ranges of two single NPs and the observed number of particles in the same size ranges of a binary mixture of these NPs.

Normality was checked using the Shapiro–Wilk test. A value of *p* ≤ 0.05 was considered statistically significant.

## 5. Conclusions

The individual aquatic toxicity assessment of the NPs in the marine microalga *H. akashiwo* demonstrated the highest toxicity of a mesoporous silicon dioxide sample with metal inclusions SMB24 (EC50, 3.6 mg/L) and sample CdS (EC50, 21.3 mg/L). The toxicity assessment of the binary mixtures of NPs revealed that the toxicity of SMB24 was reduced in the presence of CdS and ZnS by probable scavenging of additional Zn^2+^ ions and ZnO inclusions from SMB24. The tested TiO_2_ and mesoporous silicon dioxide sample without any inclusions of SMB3 demonstrated synergistic toxic action in all the combinations with the other NPs except SMB24. The used TiO_2_ NPs and the NPs in all the combinations of TiO_2_ with the other samples strongly agglomerated after 96 h of exposure in seawater. The NPs of SMB3 and the NPs in the combination of SMB3 with the other samples did not agglomerate by themselves in seawater but strongly agglomerated after the exposure with microalga cells. The supposed reason for synergistic toxic action of TiO_2_ and SMB3 combined with the other NPs is a “Trojan horse effect” which increases the bioavailability of other metal-containing NPs for aquatic organisms. Moreover, all the used NPs and their combinations agglomerated after the exposure with *H. akashiwo* compared with the similarly exposed NPs without the microalga.

The used methodology can be modified and reproduced for other types of NPs and their combinations to support a better understanding of the toxicity mechanisms and interaction between NPs and aquatic organisms. Further studies should include more species from different trophic levels, more types of NPs and their combinations, broader biomarker and target characteristic boards, omics approaches, and chronic toxicity bioassays with low doses of the toxicants. The standardization of the protocol of the mixture toxicity of NPs is one of the major issues that should be addressed to support faster study in this area.

## Figures and Tables

**Table 1:** The calculated EC10 and EC50 values of microalga growth inhibition (mg/L).

Effective Concentration	CdS	ZnS	TiO_2_	SMB3	SMB24
EC50	21.33 (18.87–23.45)	94.1 (87.4–100.7)	141.7 (133.5–151.5)	252.8 (210.4–352.2)	3.6 (3.1–4.1)
EC10	12.01 (11.67–12.35)	53.1 (43.7–63.4)	79.7 (72.4–88.4)	143.6 (125.9–180.1)	2.1 (1.9–2.2)

The 95% confidence limits are presented in parentheses.

**Table 2:** The concentrations of NPs used in the binary mixture bioassay (mg/L).

Sample	Concentration (mg/L)				
	CdS	ZnS	TiO_2_	SMB3	SMB24
CdS	12.0	0	0	0	0
ZnS	0	53.0	0	0	0
TiO_2_	0	0	79.5	0	0
SMB3	0	0	0	143.5	0
SMB24	0	0	0	0	2.1
CdS + ZnS	12.0	53.0	0	0	0
CdS + TiO_2_	12.0	0	79.5	0	0
CdS + SMB3	12.0	0	0	143.5	0
CdS + SMB24	12.0	0	0	0	2.1
ZnS + TiO_2_	0	53.0	79.5	0	0
ZnS + SMB3	0	53.0	0	143.5	0
ZnS + SMB24	0	53.0	0	0	2.1
TiO_2_ + SMB3	0	0	79.5	143.5	0
TiO_2_ + SMB24	0	0	79.5	0	2.1
SMB3 + SMB24	0	0	0	143.5	2.1

**Table 3:** The summary of single and binary mixture toxicity assessment and agglomeration of the NPs.

Sample	Microalgae Bioassay					Factors Affecting the Particle Agglomeration		
	EC50 (mg/L) or Combined Effect	Esterase Activity	Membrane Potential	ROS Generation	Cell Size	Time (96 h)	Combination	Alga
CdS	21.33	−	−	−	ns	−+	na	+
ZnS	94.1	ns	ns	++	−	−	na	+
TiO_2_	141.7	ns	ns	−	−	++−	na	+
SMB3	252.8	ns	ns	ns	−	−	na	++
SMB24	3.6	−	ns	−	−+	−+	na	+−
CdS + ZnS	ant	−	−	−	−	−	−	+
CdS + TiO_2_	syn	ns	−	−	+	++−	−	−+
CdS + SMB3	syn	−	−	−	ns	−	−	++
CdS + SMB24	ant	−	−	−	ns	−	−	+
ZnS + TiO_2_	syn	ns	ns	ns	−	++−	++−	+
ZnS + SMB3	syn	−	ns	ns	−	−	−	++
ZnS + SMB24	ant	ns	ns	++	−	−	−	+
TiO_2_ + SMB3	syn	ns	ns	−	ns	−+	−+	+
TiO_2_ + SMB24	add	+	ns	−	++	−+	−	+
SMB3 + SMB24	add	−	ns	−	ns	−	−	++

ROS = reactive oxygen species; ant - antagonistic; syn = synergistic; add = additive; ns = nonsignificant (*p* > 0.05); na = not applicable; + = increase of an estimated parameter; − = decrease of an estimated parameter; +− and −+ = applied for microalga cell size and particle size distribution and when there was simultaneous increase and decrease in different size ranges with a predominated increase and decrease in the effect, respectively; ++ = applied when an observed increase effect was significantly higher than the other one.

**Table 4:** Characteristics of the used NPs.

Sample	Average Diameter (nm)	Physical Properties	Impurities (%)	Structure Features	Synthesis or Manufacturer Information
CdS	5–9	band gap: 3.1 eV	–	Cubic crystal phase, sphere-like particles	[60]
ZnS	2.6–5.6	band gap: 4.0 eV	–	Cubic crystal phase, sphere-like particles	[60]
TiO_2_	32	BET surface area: 45 m^2^/g	>99.9% TiO_2_	Nanopowder, anatase crystal structure	Thermo Fisher GmbH, Kandel, Germany, CAS number 1317-70-0, product number 39953
SMB3	100	Pore diameter: 3.45 nm; BET surface area: 1260 m^2^/g	>99.9% SiO_2_	Mesoporous silicon dioxide, 3D structure	SMB^TM^ 3 Property, CENNANO Co., Ltd., Ulsan, Korea
SMB24	100	Pore diameter: 2.72 nm; BET surface area: 1124 m^2^/g	ZnO and Ag	Mesoporous silicon dioxide with ZnO and Ag encapsulation, 3D structure	SMB^TM^ 24 Property, CENNANO Co., Ltd., Ulsan, Korea

BET = Brunauer–Emmett–Teller technique.

**Table 5:** Microalgae cultivation conditions.

Parameters	Conditions
Temperature	20 ± 2 °C
pH	8.0 ± 0.2
Salinity	33 ± 1‰
Light intensity	300 μmol∙m-2∙s-1, cool white fluorescent
Light cycle	12:12 h light:dark
Cultivation chamber	250 mL Erlenmeyer flask
Age of test organisms	14–20 d, exponential growth phase
Initial bioassay cell density	1.2–1.4 × 10^4^ cells mL^−1^
Control/diluent water	f/2 medium/0.22 µm filtered seawater

**Table 6:** Bioassay endpoints and registration parameters.

Endpoint	Fluorescent Dye or Registered Parameter	Dye Concentration/Duration of Staining *	Instrument	Excitation Source (nm)	Emission Channel/ Band Width (nm)
Growth inhibition	PI	15 µM/10 min	CytoFLEX	488	610/20
Size	Forward scatter intensity (size calibration kit F13838 by Molecular Probes, USA)	–	CytoFLEX	488	FSC
Esterase activity	FDA	50 µM/30 min	GloMax	490	510–570
Membrane potential	DiOC_6_	7.5 µM/10 min	GloMax	490	510–570
ROS generation	H_2_DCFDA	250 µM/40 min	GloMax	490	510–570

ROS = reactive oxygen species; PI = propidium iodide; FDA = fluorescein diacetate; DiOC6, 3,3′ = dihexyloxacarbocyanine iodide; H_2_DCFDA, 2′,7′ = dichlorodihydrofluorescein diacetate. * For PI, concentration and duration of staining optimization were described in our previous work [46]. The staining parameters for FDA, DiOC_6_, and H_2_DCFDA were optimized for the GloMax microplate reader by a series of measurements, which included a broad range of concentrations and registration after every 2 min.
